# Electro-Acupuncture Alleviates Chronic Unpredictable Stress-Induced Depressive- and Anxiety-Like Behavior and Hippocampal Neuroinflammation in Rat Model of Depression

**DOI:** 10.3389/fnmol.2018.00149

**Published:** 2018-05-31

**Authors:** Na Yue, Bing Li, Liu Yang, Qiu-Qin Han, Hui-Jie Huang, Ya-Lin Wang, Jing Wang, Rui Yu, Gen-Cheng Wu, Qiong Liu, Jin Yu

**Affiliations:** ^1^Department of Integrative Medicine and Neurobiology, State Key Laboratory of Medical Neurobiology, School of Basic Medical Sciences, Institutes of Brain Science, and Collaborative Innovation Center for Brain Science, Shanghai Medical College, Fudan University, Shanghai, China; ^2^Center Laboratories, Jinshan Hospital of Fudan University, Shanghai, China; ^3^Department of Anatomy, Histology and Embryology, School of Basic Medical Sciences, Shanghai Medical College, Fudan University, Shanghai, China; ^4^Key Laboratory of Medical Imaging Computing and Computer Assisted Intervention of Shanghai, Shanghai, China

**Keywords:** depression, EA, chronic unpredictable stress, IL-1β, P2X7 receptor, glia

## Abstract

Depression is the second leading cause of disability worldwide. The effects of clinical depression may be mediated by neuroinflammation such as activation of microglia and high levels of proinflammatory cytokines in certain brain areas. Traditional Chinese medicine techniques such as electro-acupuncture (EA) are used extensively in Asia to treat mental health disorders. However, EA has not been rigorously studied in treatment of depression. This study was designed to assess the effectiveness of EA on depressive-like behavior and explore the role of hippocampal neuroinflammation in the potential antidepressant effect of EA. In this study, we used six chronic unpredictable stressors daily in a random sequence for 10 weeks. EA were performed on “Bai-Hui” (Du-20) (+) and “Yang-Ling-Quan” (GB-34, the right side; −) acupoints by an EA apparatus (HANS Electronic Apparatus, LH202H, 2/100 Hz, 0.3 mA) for 30 min once every other day for last 4 weeks. The behavior tests including open field test and forced swimming test, which are widely used to assess depressive and anxiety-like behavior were performed on the Monday and Tuesday of the eleventh week. The results showed that 10 week of chronic unpredictable stress (CUS) caused behavioral deficits in rats and neuroinflammation in hippocampus, such as increased expression of NLRP3 inflammasome components, upregulated mRNA level of IL-1β and the protein level of IL-1β mature form (p17) and activation of microglia. Moreover, 4 weeks of EA treatment significantly attenuated behavioral deficits caused by CUS. EA’s antidepressant effect was accompanied by markedly decreased expression of certain NLRP3 inflammasome components and matured IL-1β. Meanwhile, EA treatment can significantly reverse CUS-induced increases in P2X7 receptor, Iba-1, IL-18, TNFα and IL-6 expression and decreases in GFAP expression. In conclusion, EA exhibited the antidepressant effect and alleviated the hippocampal neuroinflammation. These findings may provide insight into the role of hippocampal neuroinflammation in the antidepressant effect of EA.

## Introduction

Depression is a prevalent mental health condition that affects more than 300 million people worldwide^1^, and has become the leading cause of ill health and disability across the world^1^. Depression is the most common psychiatric disorder with a high mortality and morbidity rate^2^. In the past decades, preclinical studies have demonstrated that neuroinflammation, described by the increase of inflammatory cytokines in the brain, contributed to the development of depressive behavior (Yirmiya et al., 2000; Dantzer et al., 2008; Eyre and Baune, 2012). Patients with major depressive disorder (MDD) have greater pro-inflammatory cytokines in peripheral circulation and some in brain regions, such as: interleukin-6 (IL-6), IL-1β and C-reactive protein (CRP; Howren et al., 2009; Jacoby et al., 2016). Also, animal studies demonstrate that chronic stress can lead to elevated IL-1β in several brain regions, including the hippocampus, a key area implicated in the stress response and responsible for memory and emotion (Liu et al., 2013; Pan et al., 2014). Central administration of IL-1β produces several stress-like effects and pathological changes, such as the decline of adult hippocampal neurogenesis (Green and Nolan, 2012; Green et al., 2012). Treatment with IL-1β receptor antagonist reversed chronic unpredictable stress (CUS) induced depressive—like behavior (Koo and Duman, 2008, 2009; Koo et al., 2010).

Nowadays, all available antidepressants whose action is based on the monoamine hypothesis of depression have many side effects and limited efficacies, which limits their usages and results in poor compliance. Ultimately, many patients have turned to complementary and alternative medicine (CAM), such as acupuncture. As a modern therapeutic practice of CAM, electroacupuncture (EA) combines standard acupuncture with a gentle electrical current to stimulate acupuncture points. EA has been used successfully to treat depression in China and several other countries worldwide for its efficiency and minimal adverse effects (Ulett et al., 1998; Kim et al., 2013; Guo et al., 2016; Schroeder et al., 2017). Previous clinical and preclinical studies have showed that EA relieved depressive-like behavior in patients with depression and animal models (Ulett et al., 1998; Duan et al., 2016; Li et al., 2017). However, the underlying mechanism responsible for the antidepressant-like effects of EA remains unclear. It is thought to influence neuroinflammation in preclinical studies (Guo et al., 2014). EA has been shown to reduce neuroinflammation in animal models of neuropathic pain (Li et al., 2014). Moreover, our previous articles have confirmed that EA can improve hippocampal neurogenesis and atrophy of astrocytes in animal models of depression (Liu et al., 2007; Yang et al., 2013). EA may inhibit neuroinflammation and hence produce favorable effects to hippocampal pathological changes and depressive symptoms. Hence, it would be of interest to investigate whether hippocampal neuroinflammation is responsible for the antidepressant effects of EA.

The recently emerging evidence has suggested that the nucleotide binding and oligomerization domain-like (Nod) receptor family pyrin domain-containing 3 (NLRP3) inflammasome, the best characterized member of the intracellular Nod-like receptors (NLR) family, plays a critical role in various inflammatory diseases (Bigford et al., 2013; Parajuli et al., 2013; Meng et al., 2014; Yang F. et al., 2014). The NLRP3 inflammasome is a multiprotein complex that comprises an NLRP3 receptor, an adaptor ASC (apoptosis-associated speck-like protein containing a carboxy-terminal CARD) and an effector caspase-1 (p45). Caspase-1 is cleaved into active caspase-1 (p10), which further cleaves the inactive forms of IL-1β and IL-18 (i.e., pro-IL-1β and pro-IL-18) into their mature and active forms (Bauernfeind et al., 2009; Hanamsagar et al., 2012). MDD patients exhibited an increased level of the NLRP3 inflammasome in their peripheral blood mononuclear cells (Alcocer-Gómez and Cordero, 2014). Furthermore, in LPS or chronic stress-induced rodent models of depression, rodents displayed depressive-like behavior and activation of NLRP3 inflammasome in some brain areas such as the hippocampus (Lu et al., 2014; Zhang et al., 2014), which were decreased after standard antidepressant medications (Pan et al., 2014; Du et al., 2016).

Microglia, the resident innate immune cells in the brain, play an important role in some neurodegenerative diseases (Colonna and Butovsky, 2017). Activated microglia has been regarded as a key source of local pro-inflammatory cytokines, including IL-1β, IL-18, TNFα and IL-6, driving progressive neuron damage (Song and Wang, 2011). Recent evidence suggests that NLRP3 inflammasome was only functional in mouse brain microglia but not in astrocytes (Gustin et al., 2015). Meanwhile, activation of microglial NLRP3 inflammasome mediates IL-1β-related inflammation in the prefrontal cortex of depressive rats (Pan et al., 2014). In addition, the ATP-gated trans-membrane P2X7 receptor (P2X7R) is a non-selective cation channel that contributes to the development of inflammation via the regulation of the expression and release of inflammatory cytokines (i.e., IL-1β and TNF) from microglia (Sperlagh and Illes, 2014). Moreover, P2X7R has also received specific attention for playing an essential role in NLRP3 inflammasome activation (Di Virgilio, 2007). Two recent studies have shown further evidence that antidepressant drugs can modulate central NLRP3 inflammasome activation, microglial structure and function (Pan et al., 2014; Du et al., 2016), thereby suggesting the therapeutic potential of targeting neuroinflammation to treat depression.

In this study, we sought to understand whether EA could improve behavioral deficits and decrease neuroinflammtion in rats exposed to CUS. Specifically, we examined behavioral endpoints including behavioral despair, exploration and locomotor activity, and relevant neuroinflammatory markers (NLRP3 inflammasome, proinflammatory cytokines and activation of microglia) in the hippocampus.

## Materials and Methods

### Animals

Male Sprague-Dawley rats (180–200 g) were housed 2–4 per cage in an air-conditioned (22 ± 1°C) colony room maintained under a 12 h/12 h light/dark cycle with *ad libitum* access to food and water (except when indicated). Thirty-three rats were randomly divided into four groups. Group 1 included eight healthy rats which were left undisturbed in their home cages for total 10 weeks as a normal control (Group 1: Normal); Group 2 experienced only CUS for 10 weeks (Group 2: CUS, *n* = 9); Group 3 and Group 4 was subjected to 10-week CUS and 4-weeks sham-EA or EA treatment during the last 4 weeks of CUS (Group 3: Sham-EA, *n* = 8; Group 4: EA, *n* = 8). Then, the experiment was repeated in the other 16 SD rats, which were also randomly divided into four groups and exposed to same treatment (*n* = 4 per group). This study was carried out in accordance with the National Institute of Health Guide for the Care and Use of Laboratory Animals, and the protocol was approved by Animal Ethics Committee of Shanghai Medical College, Fudan University, Shanghai, China (20160225-071).

### CUS Procedure

As previously described, SD rats were exposed to one of the six mild stressors daily in a random sequence for 10 weeks (Yang et al., 2013). Six different stressors were used in the experiment: cold swimming (swimming in 4°C water for 5 min), water deprivation (40 h), food deprivation (40 h), light-dark cycle reversal, Heat stress (40°C environment for 5 min) and shake stress (shaking cage for 30 min); see Supplementary Table S1.

### EA Delivery

**Figure 1:**
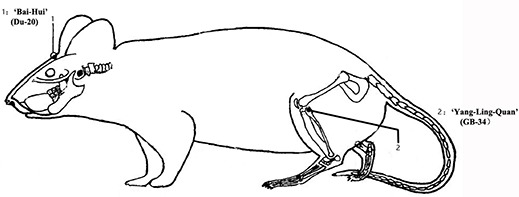
The acupuncture points of rats.

EA treatment was started in the 7th week of 10-week CUS, once every other day for 4 weeks (the latter part of the CUS period). The rats (*n* = 8–9 per group) were placed in wooden holders which restrained movement of the rat’s body but allowed relatively free head movements when treated with EA in the first experiment. However, in the repeated experiment, the rats (*n* = 4 per group) were hang up with a piece of clothes when treated with EA, staying awake, convenient and safe. The rats were in transient isoflurane anesthesia (<1 min) when they got dressed. After the rats had been acclimated the holders or clothes, EA stimulation was delivered by an EA apparatus (HANS Electronic Apparatus, LH202H, 2/100 Hz, 0.3 mA) with the electrodes connected to two acupuncture needles which were inserted into “Bai-Hui” (Du-20, located above the apex auriculate, on the midline of the head) (+) and contralateral “Yang-Ling-Quan” (GB34, located near the knee joint, anterior and inferior to the small head of the fibula, in muscle peroneus longus and brevis) (−) acupoints for 30 min (Figure 1). EA at these two acupoints produced some antidepressant effect in our previous work and other studies (Lippert, 2007; Yang et al., 2013; Li et al., 2014; Yang L. et al., 2014). Animals allocated to the Sham-EA group were subjected to the similar procedure as EA group, but no electrical current was applied to them. Animals in Normal and CUS groups were only bounded in wooden holders or in a piece of clothes without any acupuncture needles.

### Behavioral Testing

The open field test was performed in a 100 cm × 100 cm × 40 cm black Plexiglass box with a black floor as described (Redmond et al., 1997). Rats were individually placed in the center of the testing box at the beginning of the test, and then, the number of rearing behavior, the distance traveled, the center square entries and duration and the defecation in the box were recorded for 5 min and subsequently analyzed using a video-tracking system (Shanghai Mobile Datum Information Technology Company, Shanghai, China). After testing each animal, the apparatus was cleaned with 1% acetic acid to remove olfactory cues.

In the forced swimming test, individual rats were placed softly into an 18 cm diameter glass cylinder filled to 30 cm in depth with 23 ± 1°C water for 5 min. Rat’s immobility and struggling behavior during the 5 min swim session were recorded and quantified by the investigators who were unaware of group assignment. Struggling was defined as vigorous movements of the forepaws breaking the water and immobility was defined when rat floated without struggling and making only those movements necessary to keep its head above the water.

### Western Blot Analysis

The hippocampi of rats (*n* = 4 per group, from the first experiment) were ultrasonically disrupted in RIPA buffer (50 mM Tris (pH 7.4), 150 mM NaCl, 1% Triton X-100, 1% sodium deoxycholate, 0.1% sodium dodecyl sulfonate, sodium orthovanadate, sodium fluoride, ethylene diamine tetraacetic acid, leupeptin, Thermo Scientific) with protease inhibitors (PMSF, Beyotime) followed by centrifugation at 12,000× *g* for 20 min. Then the total protein level in the supernatants was measured using the Pierce BCA Protein Assay Kit (Thermo Scientific, Rockford IL, USA). Protein samples were separated on 12% acrylamide gels which were cut into two parts about at 45 kD band according to the protein ladders and interest proteins, then transferred to PVDF membrane (0.2 and 0.45 μm, respectively). After blocking with 5% nonfat milk in tris-buffered-saline with tween (TBST; 20 mM Tris-HCl, pH 7.5, 150 mM NaCl, and 0.05% Tween-20) for 2 h at 4°C, the membrane was blotted respectively with antibodies against Iba-1 (1:1000, Wako) and GAPDH (1:10,000, Kang Cheng) as well as P2X7R (1:200, Santa Cruz) and GFAP (1:1000, Thermo Scientific) in sequence. For detecting the IL-1beta, ASC, Caspase-1 levels, the gels were cut into two parts about at 25 kD band and then transferred to PVDF membrane (0.2 and 0.45 μm, respectively), and blotted with antibodies against IL-1β (1: 1000, R&D System) or caspase 1 (1:200, Santa Cruz) and GAPDH (1:10,000, Kang Cheng) in sequences. Using the same procedure, we also detected NLRP3 expression levels using Cryopyrin (NLRP3, 1:200, Santa Cruz) and GAPDH (1:10,000, Kang Cheng) antibodies. And primary antibody incubation was performed at 4°C overnight.

After the blots were washed in TBST five times, the secondary antibodies (1:10,000, Earthox) were incubated for 1 h at room temperature. Western blot images were captured on an Image Quant LAS4000 mini image analyzer (GE Healthcare, Buckinghamshire, UK), and the band levels were quantified using Image J software (NIH, Bethesda, MD, USA).

### Quantitative Real-Time RT-PCR

The hippocampi of rats (*n* = 4 per group from the first experiment) were homogenized and RNA was isolated using TRIzol reagent (Invitrogen) according to the manufacturer’s instructions. The quality and quantity of the extracted RNA in each tissue was examined with spectrophotometry (Beckman DU7500). Equal amounts of RNA (2 μg/sample) isolated from the each hippocampi was reacted with M-MLV reverse transcriptase (iScriptTM cDNA Synthesis Kit, Bio-Rad, CA, USA) to generate cDNA in the following reaction: 5 μl of 5× M-MLV Reverse Transcriptase Reaction Buffer which included, in the final concentration, 1 μl of 0.5 ug/μl Oligo(dT)15 Primer (Bio-Rad), 2 μl of 10 mM dNTP Mix (Bio-Rad), 1 μl of 200 U/μl M-MLV reverse transcriptase (Bio-Rad, CA, USA), and 0.5 μl of 40 U/μl RNase inhibitor (Bio-Rad, CA, USA). Each reaction was then incubated at 37°C for 1 h. Equal amounts of cDNA (2 μl) were then used for subsequent PCR using iTap SYBR Green Master Mix (Bio-Rad, CA, USA). The 2^−ΔΔCt^ method [ΔCT = (Ct_target_ − Ct_GAPDH_)] was then used to convert 1CT values to mRNA fold changes relative to the control group. The mRNA levels of the targets were normalized with glyceraldehyde-3-phosphatedehydrogenase (GAPDH) mRNA level to exclude effects of varying RNA amounts.

Please see Supplementary Table S2 for oligonucleotide primers specific for rat.

### Immunohistochemical Analysis

The brains of rats (*n* = 4 per group from the second experiment) were removed and post fixed in 4% PFA at 4°C overnight and immersed in 20% sucrose (4% PFA as solvent) followed by 30% sucrose (in 0.1 M PBS). Thirty micrometer thick sections (CM1850, Leica Microsystems, Wetzlar, Germany) were blocked in 2% (wt/vol) BSA (Sigma) and then exposed overnight to the following primary antibody mixtures: anti-GFAP(Thermo, 1:1000) or anti-Iba-1(Wako, 1:1000) at 4°C. After washed five times in PBS, sections were incubated with secondary antibodies (donkey-anti-mouse, Alexa 594 conjugated, 1:1000, Invitrogen, USA; donkey anti rabbit, Alexa 594 conjugated, 1:1000, Invitrogen, USA; Hoechst, 1:1000, Beyotime, China) for 1 h at room temperature in the dark. These sections were rinsed in PBS five times 5 min each and cover slipped in the dark. Sections were imaged at 20× and 40× for analysis, using excitation wavelengths of 633 nm (blue Cy5 labeling), 543 nm (red Cy3 immunofluorescence).

### Statistical Analyses

All data are analyzed using SPSS 16.0 (SPSS Inc., Chicago, IL, USA) and expressed as the mean ± standard error. The data collection and analysis were performed independently by two experimenters. The statistical significance of differences between groups was analyzed using one-way analysis of variance (ANOVA) according to the factors introduced in the experimental design. Where *F* ratios were significant, *post hoc* comparisons were made using the Tukey* post hoc* test. Significance levels were set at *p* < 0.05.

## Results

### EA Treatment Ameliorates Depressive-Like Behavior Induced by Chronic Unpredictable Stress (CUS)

**Figure 2:**
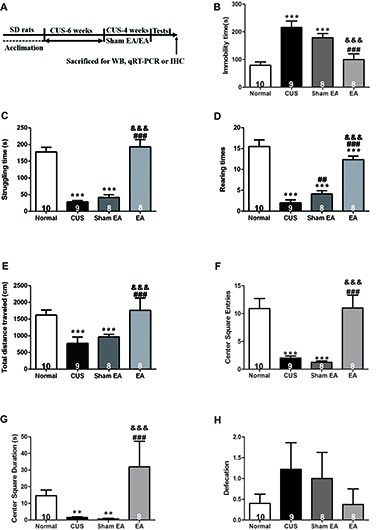
Electro-Acupuncture (EA) alleviates depressive-like behavior induced by chronic unpredictable stress (CUS) in Sprague-Dawley (SD) rats. **(A)** Experimental paradigm. Forty-nine male SD rats were randomly divided into four groups and treated as shown above. Behavioral indicators were then assessed, including. **(B)** Immobility time, **(C)** Struggling time in forced swimming test (FST). **(D)** Rearing numbers. **(E)** Total distance traveled in open field test (OFT). **(F)** Center square entries. **(G)** Center square duration and **(H)** Defecation. All data are expressed as the mean ± SEM (*n* = 8–10 per group). ** *p* < 0.01, ***p < 0.001, compared with Normal group; ^##^*p* < 0.01, ^###^*p* < 0.001, compared with CUS group; ^&&&^*p* < 0.001, compared with Sham EA group.

In this study, the influence of long-term EA treatment on depressive-like behavior was evaluated. Experimental paradigm used in the experiment is depicted in Figure 2A. Except for rats in the Normal group, the other SD rats were exposed to CUS for 10 weeks, and treated with EA, Sham-EA every other day or no treatment during the last 4 weeks of CUS. Ten weeks of CUS exposure induced significantly depressive and anxiety-like behavior, indicated by more immobility time (*F*_(3,29)_ = 13.311, *p* < 0.001) and less struggling behavior (*F*_(3,29)_ = 30.946, *p* < 0.001) in FST (Figures 2B,C), less rearing behavior (*F*_(3,29)_ = 28.364, *p* < 0.001), shorter distance traveled (*F*_(3,29)_ = 8.207, *p* < 0.001), less center square entries (*F*_(3,29)_ = 13.899, *p* < 0.001) and less center square duration (*F*_(3,29)_ = 3.008, *p* < 0.01) in OFT (Figures 2D–G). Although CUS exhibited a tendency to increase the defecation of rats in OFT, there are no significant difference between CUS and Normal group (*F*_(3,29)_ = 0.726, *p* = 0.0513, Figure 2H).

After 4 weeks of EA treatment, rats exhibited less immobility time (*F*_(3,29)_ = 13.311, *p* < 0.001) and more struggling behavior (*F*_(3,29)_ = 30.946, *p* < 0.001) in FST as compared to rats that were only exposed to CUS or treated with Sham-EA simultaneously, which indicated that EA alleviated behavioral despair (Figures 2B,C). Furthermore, rats treated with EA also exhibited more rearing behavior (*F*_(3,29)_ = 28.364, *p* < 0.001) and traveled longer distances (*F*_(3,29)_ = 8.207, *p* < 0.001), spent more time on the center square (*F*_(3,29)_ = 3.008, *p* < 0.001), more frequently entered the central square (*F*_(3,29)_ = 13.899, *p* < 0.001) in OFT (Figures 2D–G), which indicated that EA increased exploration and the locomotor activity in CUS rats as well. But there is no significant difference in defecation among all four groups (*F*_(3,29)_ = 0.726, *p* = 0.198, Figure 2H). These results suggest EA treatment could reverse the CUS-induced depressive- and anxiety-like behavior, substantiating the evidence for antidepressant-like and anxiolytic effects of EA treatment.

### CUS-Induced Increase in IL-1β and NLRP3 Inflammasome in the Hippocampus Is Reversed by EA Treatment

**Figure 3:**
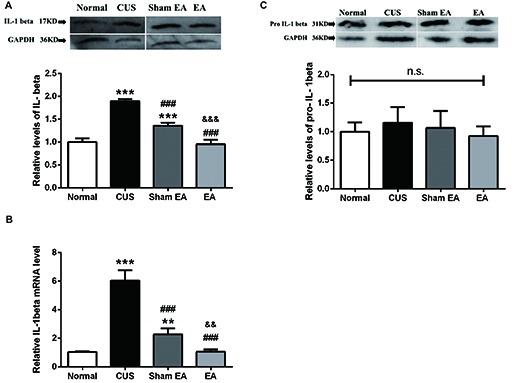
EA downregulates mRNA level of IL-1beta and protein level of mature IL-1beta (p17), which were upregulated by CUS in hippocampi of rats. After behavior tests, rats’ hippocampi were collected and analyzed for protein level of **(A)** mature IL-1β (p17), **(C)** pro-IL-1β (p35), and **(B)** the mRNA level of IL-1β using western blotting and quantitative PCR. Results are expressed as the mean ± SEM (*n* = 4 per group. **p < 0.01, ***p < 0.001, compared with Normal group; ^###^*p* < 0.001, compared with CUS group; ^&&^*p* < 0.01, ^&&&^*p* < 0.001, compared with Sham EA group; n.s, not significant.

Western blotting analysis showed that the levels of matured IL-1β (p17) in the hippocampus were significantly increased in rats belonging to the CUS and Sham-EA groups, respectively, as compared to the Normal group (*F*_(3,12)_ = 137.2, *p* < 0.001, Figure 3A). EA treatment significantly decreased the level of matured IL-1β as compared with the Sham-EA group or CUS group (*p* < 0.001, Figure 3A). Likewise, EA also downregulated the mRNA level of IL-1β which increased in the CUS group (*F*_(3,12)_ = 122.5, *p* < 0.001, Figure 3B). Interestingly, although there was no significant difference in the protein level of pro-IL-1β (p31) in the hippocampus across all experimental groups (*F*_(3,12)_ = 0.7327, *p* = 0.5522, Figure 3C), the relative mRNA level of IL-1β was significantly enhanced by exposure to CUS (*p* < 0.001, Figure 3B). The results hinted that CUS may also upregulate the cleavage of IL-1β except for the transcription of IL-1β.

**Figure 4:**
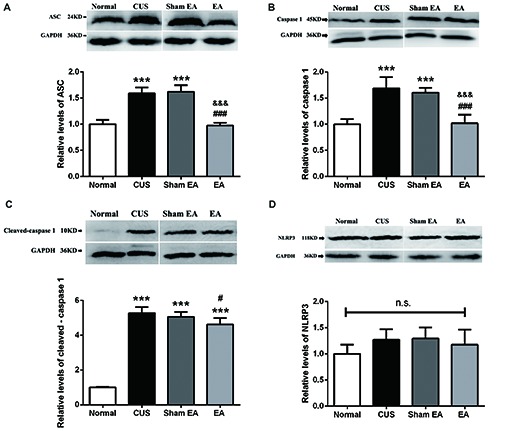
EA decreases the protein level of ASC, caspase 1 (p45) and cleaved-caspase 1 (p10) but not NLRP3 in hippocampi of CUS rats. After behavior tests, rats’ hippocampi were collected and analyzed for levels of **(A)** ASC, **(B)** caspase 1 (p45), **(C)** cleaved-caspase 1 (p10) and **(D)** NLRP3 using western blotting. Results are expressed as the mean ± SEM (*n* = 4 per group). ***p < 0.001, compared with Normal group; ^#^*p* < 0.05, ^###^*p* < 0.001 compared with CUS group; ^&&&^ compared with Sham-EA group; n.s., not significant.

Thus, NLRP3 inflammasome components, including NLRP3, ASC and caspase-1, in the hippocampus were also evaluated by western blotting. As shown in Figure 4A, the hippocampal ASC protein level significantly increased in the CUS and Sham-EA groups as compared to the Normal group (*F*_(3,12)_ = 52.88, *p* < 0.001). The levels of caspase-1 (p45; *F*_(3,12)_ = 23.76, *p* < 0.001) and cleaved-caspase-1 (active caspase-1, p10; *F*_(3,12)_ = 188.8, *p* < 0.001) in the hippocampus were also significantly increased in the CUS and Sham-EA groups as compared to the Normal group (Figures 4B,C). Simultaneously, EA treatment showed a significant decrease in hippocampal ASC protein expression (*p* < 0.001, Figure 4A) as well as caspase-1 (p45; *p* < 0.001, Figure 4B) as compared to the Sham-EA group and CUS group. The level of cleaved-caspase-1 (p10) in the hippocampus also exhibited a slight decrease in the EA group as compared to the CUS group (*p* < 0.05, Figure 4C). However, NLPR3 protein expression in the hippocampus did not differ significantly among the four experimental groups (*F*_(3,12)_ = 1.425, *p* = 0.2838, Figure 4D).

### CUS-Induced Changes of P2X7R, Iba-1 and GFAP Expression in the Hippocampus Is Regulated by EA Treatment

**Figure 5:**
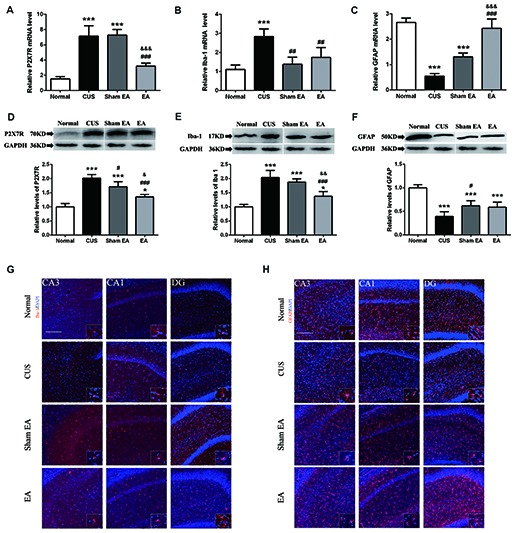
Effect of EA on gene and protein expression of P2X7R, GFAP and Iba-1 in hippocampi of CUS rats. The relative levels of protein corresponding to **(A)** P2X7R, **(B)** GFAP and **(C)** Iba-1 were assessed using an immunoblotting method. Results were normalized to GAPDH. Levels in Normal rats were arbitrarily assigned a value of 1.0. The relative levels of mRNAs encoding for **(D)** P2X7R, **(E)** GFAP and **(F)** Iba-1 were assessed using quantitative PCR. Results were normalized to GAPDH. All values are the means ± SEM (*n* = 4). *p < 0.05 and ***p < 0.001, compared with Normal group. ^#^*p* < 0.05, ^##^*p* < 0.01, ^###^*p* < 0.001, compared with CUS group. **(G,H)** Immunofluorescence staining of hippocampal sections. GFAP and Iba-1 expression in the CA1, CA3, dentate gyrus (DG) of hippocampus detected by immunofluorescence in Normal rats and rats exposed to CUS with/without EA or Sham EA treatment. Representative images show GFAP/Iba-1 (red) and DAPI (blue). ^&^*p* < 0.05, ^&&^*p* < 0.01, ^&&&^*p* < 0.001, compared with Sham EA group.

Given that P2X7R is one of the vital factors for NLRP3 inflammasome activation (Lu et al., 2014), we also observed the expression of P2X7R in the hippocampus. As shown in Figures 5A,D, the hippocampal P2X7R mRNA (*F*_(3,12)_ = 49.35, *p* < 0.001) and protein levels (*F*_(3,12)_ = 45.26, *p* < 0.001) were significantly increased in the CUS and Sham-EA groups as compared to the Normal group. EA treatment also significantly reduced the hippocampal P2X7R mRNA (*p* < 0.001) and protein levels (*p* < 0.001) as compared to the CUS or Sham-EA groups.

**Figure 6:**
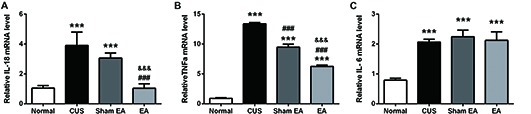
EA downregulates mRNA level of IL-18, TNFα, which were upregulated by CUS in hippocampi of rats. After behavior tests, rats’ hippocampi were collected and analyzed for mRNA level of **(A)** IL-6, **(B)** IL-18, and **(C)** TNF α using quantitative PCR. Results are expressed as the mean ± SEM (*n* = 4 per group. ***p < 0.001, compared with Normal group; ^###^*p* < 0.001, compared with CUS group; ^&&&^*p* < 0.001, compared with Sham EA group.

A primary source of CNS inflammatory cytokines is the activated microglia cells (Meng et al., 2014). Here, we used the microglia marker, Iba1, to evaluate microglia activation. As shown in Figures 5B,E,G, after exposure to CUS, the mRNA (*F*_(3,12)_ = 14.91, *p* < 0.001) and protein levels (*F*_(3,12)_ = 32.89, *p* < 0.001) of hippocampal Iba1 in the hippocampus were significantly increased as compared to the Normal group. Meanwhile, EA treatment significantly decreased the mRNA and protein level of Iba1 in the hippocampus (Figures 5B,E). Interestingly, Sham-EA treatment also significantly downregulated the mRNA level of Iba1 (*F*_(3,12)_ = 14.91, *p* < 0.01) but not the protein level (*F*_(3,12)_ = 14.91, *p* = 0.062) as compared with the CUS group (Figures 5B,E). In addition to IL-1β, we also assessed the transcription of other proinflammatory cytokines, such as, IL-18, TNF1β and IL-6 in the hippocampus, using real-time quantitative PCR. The results exhibited that exposure to CUS induced significant upregulation of the mRNA level of IL-18 (*F*_(3,12)_ = 32.65, *p* < 0.001), TNF1x003B1; (*F*_(3,12)_ = 12.51, *p* < 0.001) and IL-6 (*F*_(3,12)_ = 52.28, *p* < 0.001) in the hippocampus (Figures 6A–C). Meanwhile, the mRNA level of IL-18, TNF1x003B1; but not IL-6 in the hippocampus was cut down significantly in the EA group as compared with the CUS group and Sham-EA group (Figures 6A–C). The results give further support to the supposition that CUS-induced neuroinflammation in the hippocampus was alleviated by EA treatment.

In addition, astrocytes have been regarded as the source of certain proinflammatory cytokines. However, there is increasing evidence of astrocytic atrophy or dysfunction in depressive disorders. Hence, we also evaluated astrocyte levels using the astrocyte marker, GFAP. As shown in Figures 5C,F,H, both GFAP mRNA (*F*_(3,12)_ = 78.82, *p* < 0.001) and protein expression (*F*_(3,12)_ = 27.75, *p* < 0.001) in the hippocampus were significantly decreased in the CUS group as compared to the Normal group. As compared to the CUS group, Sham-EA treatment significantly increased hippocampal GFAP protein and the mRNA level (Figures 5C,F). EA Treatment only significantly increased the GFAP mRNA level, but not the protein level, when compared to the Sham-EA and CUS groups (Figures 5C,F).

## Discussion

The present study demonstrated that 10 weeks of CUS-induced depressive- and anxiety-like behavior, which are described as a dramatic increase in the immobility time in FST and a significant decrease in the rearing frequency and total distance traveled as well as less central square entries and duration in OFT (Figure 2H). Meanwhile, EA treatment ameliorated the depressive- and anxiety-like behavior. In addition, EA inhibited the increase of the level of hippocampal IL-1β protein and its’ convertase (active caspase 1) caused by CUS. Likewise, the protein levels of ASC and pro-caspase 1 p45 (inactive caspase-1), two important components of NLRP3 inflammasome, which significantly increased in CUS rats, were also diminished by EA treatment. Moreover, CUS caused the significant upregulation of the P2X7R mRNA and protein levels, which is accompanied by microglia activation and astrocytic atrophy. EA treatment also alleviated this CUS-induced hippocampal pathology.

### CUS Induces Depressive-Like Behavior and EA Exhibits the Antidepressant-Like Effect

CUS induced in animal models is often regarded as one of the strongest animal models of depression because of its good predictive validity (Henn and Vollmayr, 2005), face validity and construct validity. After exposure to CUS, the mice or rats will exhibit some depressive-like behavior, such as behavioral despair, anhedonia, less exploration and less locomotion (Hazra et al., 2017; Jett et al., 2017). In our experiments, we also evaluated the depressive-like behavior in an open field test (OFT) and forced swimming test (FST). The present study showed that CUS induced an obvious decrease in exploration and locomotion in OFT and less struggling and more immobile behavior in FST. Like many antidepressants (Liu et al., 2008; Mutlu et al., 2012), EA also significantly relieved the depressive-like behavior induced by CUS. The results underpin the antidepressant-like effects of EA consistent with the previous studies (Liu et al., 2007; Duan et al., 2016; Li et al., 2017).

### CUS Upregulates IL-1β and NLRP3 Inflammasome

Evidence is increasing that psychological and physical stressors could activate immune and inflammation processes, contributing to depressive symptoms (Iwata et al., 2013). Proinflammatory cytokine IL-1β in some brain areas of the limbic system, such as the prefrontal cortex and hippocampus of depressive rats, was implicated in the pathophysiology of depression (Pan et al., 2014). The result that exposure to CUS increased hippocampal matured IL-1β (p17) is consistent with these findings. Recent research has also indicated that activation of NLRP3 inflammasome signaling, a pivotal mediator of IL-1β function (Haneklaus et al., 2013), contributes to depression (Iwata et al., 2016). Additionally, several other studies have also reported the change of NLRP3 inflammasome components in rodents exposed to CUMS, LPS stimulus or estrogen deficiency (Lu et al., 2014; Zhang et al., 2014; Xu et al., 2016). In their studies, they observed the overexpression of certain components of NLRP3 inflammasome, such as NLRP3, ASC and caspase-1. In accordance with their results, our results also showed an increase in the expression of ASC, one of the components of NLRP3 inflammasome and caspase-1 (p45 and p10), the effector and the product of NLRP3 inflammasome, respectively, after exposure to CUS. Taken together, it implies that CUS upregulates the expression of NLRP3 inflammasome components and induces the activation of NLRP3 inflammasome, indicated by the increase of caspase-1 (p10). Additionally, the other important question for the field is the implication of inflammasome in antidepressant-like effect of EA. Our results exhibited that EA significantly inhibited the upregulation of components and products of NLRP3 inflammasome. In accordance with our results, other studies have indicated that the classical antidepressant, fluoxetine, and some monomeric compounds extracted from the traditional Chinese herb, such as L-Menthone and Icariin, can inhibit the activation or upregulation of NLRP3 inflammasome as well as confer their antidepressant effects (Xue et al., 2015; Du et al., 2016).

### EA Reversed CUS-Induced IL-1β-Related Microglia Activation Which May Be Mediated by P2X7-NLRP3 Signaling

Two recent articles from the Ronald S. Duman laboratory and our group have indicated that extracellular ATP-P2X7R signaling may mediate the stress-induced neuroinflammation, possibly via NLRP3 inflammasome-dependent IL-1β mature and microglia activation (Iwata et al., 2016; Yue et al., 2017). The experiment indicated that chronic accumulation of stress (10 weeks) further induced upregulation of P2X7R, IL-1beta and microglia activation. In addition, NLPR3 and P2X7R primarily localized in microglia provide further support for a tight relationship between P2X7R, NLRP3 and microglia activation (Gustin et al., 2015). Moreover, the IL-1beta mature and release was regarded as the key marker of proinflammatory activation of microglia (Song and Wang, 2011). Likewise, activated microglia was taken as the source of local synthesized cytokines in the brain (Song and Wang, 2011; Harry and Kraft, 2012). However, the research focus on microglia in depression presented contradictory results (Yirmiya et al., 2015; Santos et al., 2016). The results exhibited that CUS induced the increase of mature IL-1β, the upregulation of P2X7R, NLRP3 inflammasome and microglia activation, which raises the possibility that upregulation of P2X7R-NLRP3 inflammasome signaling may be correlated with IL-1β-related microglia activation in CUS rats. The above discussion further raises another important question relating to the implication of P2X7R and microglia activation in the effect of EA and other antidepressants. A previous study in which trifluoperazine and paroxetine suppressed P2X7-mediated IL-1β secretion from lipopolysaccharide (LPS)-primed human CD14+ monocytes has shed some light on the question (Dao-Ung et al., 2015). In addition, our previous research has confirmed that knockout P2X7R (P2X7-null or P2X7–/– mice) displayed an antidepressant phenotype after exposure to CUS. Simultaneously, the antagonist of P2X7R also can impede the depressive-like behavior induced by CUS (Iwata et al., 2016; Yue et al., 2017). All of these results give more support to the hypothesis that downregulation of P2X7R expression may mediate the antidepressant effects of EA. Interestingly, sham-EA treatment also diminished the mRNA and mature form of IL-1β like EA, although to a less extent. In clinic, acupuncture, by sticking needles into skin to some specific acupoints with some manual operation but not electric current also has efficacy on many diseases to a certain extent like EA (Li et al., 2012; Ma et al., 2012; Zhang et al., 2017). Sham-EA may be regarded as a low-intensity form of therapeutic needling which also have a little efficacy on some diseases, such as pain (Adrian White, 2009; White and Cummings, 2009). S Consistent with these findings, the results also shed some light on the relieving effect of Sham-EA on neuroinflammation, especially, IL-1β expression and mature in hippocampus.

### EA Reversed CUS-Induced Microglia Activation and Astrocytic Atrophy

As is common knowledge, microglia is the main source of pro-inflammatory cytokines in the brain (Harry and Kraft, 2012). The pro-inflammatory cytokines are suggested to be involved in the pathophysiology of MDD (Yirmiya et al., 2000; Dantzer et al., 2008; Song and Wang, 2011; Eyre and Baune, 2012). The activation of microglia may have detrimental effects on neurons by expressing and synthesizing pro-inflammatory cytokines such as IL-1β, which induces neuro-inflammation and eventually induces the death of neurons under these conditions (Brown and Vilalta, 2015; Dao-Ung et al., 2015). In our present study, we showed that CUS induced the dramatic activation of microglia, which was in accordance with the previous studies indicating that diversiform stress including: environmental, psychological and chronic stress activated microglia (Hinwood et al., 2012; Liu et al., 2015; McKim et al., 2016). However, EA treatment did not significantly alleviate the microglial activation in the hippocampus, although the microglia activation was slightly inhibited in the EA group, which implies that EA might also regulate other glia cells. Interestingly, our present results showed that EA treatment could reverse astrocytic atrophy induced by CUS in mRNA levels. Increasing evidence has unmasked the controversy that numerical alterations of astrocytes in the front limbic systems are tightly connected with depression, as implied in post-mortem studies of patients with MDD (Wang et al., 2017). Many animal studies also indicated that psychological and physiological stress can induce the dysfunction or atrophy of astrocytes (Wilhelmsson et al., 2006; Gosselin et al., 2009; Liu et al., 2009; Zhang et al., 2015; Cobb et al., 2016). The present results provide insight into the role of astrocytic atrophy in depression and the antidepressant effect of EA.

## Author Contributions

JY, NY, BL and G-CW conceived and designed the experiments. NY performed the experiments. NY, LY, Y-LW, Q-QH, H-JH, JW and RY analyzed the data. All of the authors discussed the results. JY, QL and NY wrote and modified the manuscript. All authors reviewed the manuscript.

## Conflict of Interest Statement

The authors declare that the research was conducted in the absence of any commercial or financial relationships that could be construed as a potential conflict of interest.

## Footnotes

^1^http://www.who.int/mediacenter/factsheets/fs369/en/

^2^www.who.int
