# Supplementary material for: Electro-Acupuncture Alleviates Chronic Unpredictable Stress-Induced Depressive- and Anxiety-Like Behavior and Hippocampal Neuroinflammation in Rat Model of Depression
Source: Front Mol Neurosci. 2018 May 31;11:149. doi: 10.3389/fnmol.2018.00149 (PMC6007169; doi:10.3389/fnmol.2018.00149)
Supplement: Supplementary file 1 [file Table_1.DOCX]

Table.1 The Chronic unpredictable stress procedure

| **Week** | **Monday** | **Tuesday** | **Wednesday** | **Thursday** | **Friday** | **Saturday** | **Sunday** |
| --- | --- | --- | --- | --- | --- | --- | --- |
| 1 | 8:00  Heat stress  40°C, 5min | 8：00 (Tue) - 24：00 (Wed)  water deprivation  40h | | 8:00  cold swimming  4°C, 5min | 8:00  shake stress  30min | 7:00 (Sat) - 7:00 (Sun)  light-dark cycle reversal  24h | 8:00  Heat stress  40°C, 5min |
| 2 | 8：00 (Mon) - 24：00 (Tue)  food deprivation  40h | | 8:00  cold swimming  4°C, 5min | 7:00 (Thu) - 7:00 (Fri)  light-dark cycle reversal  24h | 8:00  Heat stress  40°C, 5min | 17:00  shake stress  30min | 8:00 (Sun)- 24:00 (Mon)  water deprivation  40h |
| 3 | 8:00 (Sun)- 24:00 (Mon)  water deprivation  40h | 8:00  shake stress  30min | 7:00 (Wed) - 7:00 (Thu)  light-dark cycle reversal  24h | 8：00 (Tus) - 24：00 (Wed)  water deprivation  40h | | 8:00  cold swimming  4°C, 5min | 7:00 (Sun) - 7:00 (Mon)  light-dark cycle reversal  24h |
| 4 | 8:00  shake stress  30min | 8:00  Heat stress  40°C, 5min | 8：00 (Wed) - 24：00 (Thu)  food deprivation  40h | | 8:00  cold swimming  4°C, 5min | 7:00 (Sat) - 7:00 (Sun)  light-dark cycle reversal  24h | 8:00  Heat stress  40°C, 5min |
| 5 | 7:00 (Mon) - 7:00 (Tue)  light-dark cycle reversal  24h | 8:00  cold swimming  4°C, 5min | 8:00  Heat stress  40°C, 5min | 8:00  shake stress  30min | 8：00 (Fri) - 24：00 (Sat)  water deprivation  40h | | 17:00  cold swimming  4°C, 5min |
| 6 | 8：00 (Fri) - 24：00 (Sat)  water deprivation  40h | | 7:00 (Wed) - 7:00 (Thu)  light-dark cycle reversal  24h | 8：00 (Thu) - 24：00 (Fri)  food deprivation  40h | | 8:00  cold swimming  4°C, 5min | 7:00 (Sun) - 7:00 (Mon)  light-dark cycle reversal  24h |
| 7 | 8:00  Heat stress  40°C, 5min | 8:00  cold swimming  4°C, 5min | 8：00 (Wed) - 24：00 (Thu)  water deprivation  40h | | 7:00 (Fri) - 7:00 (Sat)  light-dark cycle reversal  24h | 8:00  Heat stress  40°C, 5min | 8:00  shake stress  30min |
| 8 | 8:00  cold swimming  4°C, 5min | 7:00 (Tue) - 7:00 (Wed)  light-dark cycle reversal  24h | 8:00  shake stress  30min | 7:00 (Thu) - 7:00 (Fri)  light-dark cycle reversal  24h | 8:00  Heat stress  40°C, 5min | 8：00 (Sat) - 24：00 (Sun)  food deprivation  40h | |
| 9 | 7:00 (Mon) - 7:00 (Tue)  light-dark cycle reversal  24h | 8:00  shake stress  30min | 8:00  Heat stress  40°C, 5min | 7:00 (Thu) - 7:00 (Fri)  light-dark cycle reversal  24h | 8：00 (Fri) - 24：00 (Sat)  water deprivation  40h | | 8:00  cold swimming  4°C, 5min |
| 10 | 8:00  shake stress  30min | 7:00 (Tue) - 7:00 (Wed)  light-dark cycle reversal  24h | 8：00 (Wed) - 24：00 (Thu)  food deprivation  40h | | 8:00  cold swimming  4°C, 5min | 8:00  shake stress  30min | 8:00  Heat stress  40°C, 5min |
| 11 | Open Field Test | Forced Swimming Test |  | |  |  |  |
