# Supplementary material for: Electro-Acupuncture Alleviates Chronic Unpredictable Stress-Induced Depressive- and Anxiety-Like Behavior and Hippocampal Neuroinflammation in Rat Model of Depression
Source: Front Mol Neurosci. 2018 May 31;11:149. doi: 10.3389/fnmol.2018.00149 (PMC6007169; doi:10.3389/fnmol.2018.00149)
Supplement: Supplementary file 2 [file Table_2.docx]

Table.2 Oligonucleotide primers used in qRT PCR

| Sequence ID (Rattus) | Orientation | Sequence (5’>3’) |
| --- | --- | --- |
|  |  |  |
| IL-1beta | Forward | ATGAGAGCATCCAGCTTCAAATC |
|  | Reverse | CACACTAGCAGGTCGTCATCATC |
| P2rx7 | Forward | CTGCCTCCCGTCTCAACTAC |
|  | Reverse | GCCTCTCTGGATAGCACGAT |
| GFAP | Forward | GAGATGATGGAGCTCAATGACC |
|  | Reverse | TGGATCTCCTCCTCCAGCGA |
| Iba-1 | Forward | TGCGCAAGAGATCTGCCATC |
|  | Reverse | ACCAGTTGGCTTCTGGTGTT |
| IL-18 | Forward | ATATCGACCGAACAGCCAAC |
|  | Reverse | TGGCACACGTTTCTGAAAGA |
| TNF-α | Forward | CGAGATGTGGAACTGGCAGA |
|  | Reverse | CTACGGGCTTGTCACTCGA |
| IL-6 | Forward | GACAAAGCCAGAGTCCTTCA |
|  | Reverse | ACTAGGTTTGCCGAGTAGAC |
| GAPDH | Forward | CCCTTCATTGACCTCAACTAC |
|  | Reverse | CTTCTCCATGGTGGTGAAGAC |
